# Supportive methods for childhood acute lymphoblastic leukemia then and now: A compilation for clinical practice

**DOI:** 10.3389/fped.2022.980234

**Published:** 2022-09-12

**Authors:** Alexandra Podpeskar, Roman Crazzolara, Gabriele Kropshofer, Petra Obexer, Evelyn Rabensteiner, Miriam Michel, Christina Salvador

**Affiliations:** ^1^Division of Hematology and Oncology, Department of Pediatrics I, Medical University of Innsbruck, Innsbruck, Austria; ^2^Department of Pediatrics II, Medical University of Innsbruck, Innsbruck, Austria; ^3^Division of Cardiology, Department of Pediatrics III, Medical University of Innsbruck, Innsbruck, Austria

**Keywords:** acute lymphoblastic leukemia, children, immunotherapy, molecular therapy, supportive therapy

## Abstract

Survival of childhood acute lymphoblastic leukemia has significantly improved over the past decades. In the early years of chemotherapeutic development, improvement in survival rates could be attained only by increasing the cytostatic dose, also by modulation of the frequency and combination of chemotherapeutic agents associated with severe short- and long-time side-effects and toxicity in a developing child's organism. Years later, new treatment options have yielded promising results through targeted immune and molecular drugs, especially in relapsed and refractory leukemia, and are continuously added to conventional therapy or even replace first-line treatment. Compared to conventional strategies, these new therapies have different side-effects, requiring special supportive measures. Supportive treatment includes the prevention of serious acute and sometimes life-threatening events as well as managing therapy-related long-term side-effects and preemptive treatment of complications and is thus mandatory for successful oncological therapy. Inadequate supportive therapy is still one of the main causes of treatment failure, mortality, poor quality of life, and unsatisfactory long-term outcome in children with acute lymphoblastic leukemia. But nowadays it is a challenge to find a way through the flood of supportive recommendations and guidelines that are available in the literature. Furthermore, the development of new therapies for childhood leukemia has changed the range of supportive methods and must be observed in addition to conventional recommendations. This review aims to provide a clear and recent compilation of the most important supportive methods in the field of childhood leukemia, based on conventional regimes as well as the most promising new therapeutic approaches to date.

## Introduction

Multi-agent risk-adapted chemotherapy regimens and the incorporation of multicenter studies have efficiently improved outcomes for children with acute lymphoblastic leukemia (ALL) over the past two decades (1, 2). The recognition of response characteristics that identify patients at risk for treatment failure resulted in the practice of augmenting treatment intensity and drug dosage adjustment based on personalized risk stratifications (3, 4). Thanks to these advances, patients with high-risk criteria or relapse can nowadays be treated efficiently using conventional second-line regimes (5). Nevertheless, despite improvements in survival rates, a subset of patients with poor prerequisites or high-risk genotypes still pose a major challenge for pediatric oncologists. New insights into the genetic heterogeneity of ALL have spurred the development of drugs to target molecular lesions that promote the survival of aberrant leukemic cells and thus paved the way to fighting the most resistant phenotypes (3, 4, 6). In patients with Philadelphia (Ph)-positive ALL, the development of tyrosine-kinase-inhibitors (TKI) was a breakthrough for previously poor treatment outcomes. Innovative therapeutic agents such as autologous chimeric antigen receptor (CAR) T-cell therapy or the bispecific antibody blinatumomab have emerged as effective treatment strategies for such difficult-to-treat leukemias. These novel targeted drugs show great promise but are also associated with unique and often severe toxicities and side-effects (7–13). Advanced supportive care (SC) methods could be paramount to ameliorate outcomes further, reduce side-effects, and ensure that patients derive the most benefit from ALL therapy (14, 15). Considering the constant development of oncological therapies, the management of complex new side-effects implies a constant adaptation of supportive care measurements. Nowadays there is a flood of publications on supportive methods in the oncological literature (even in the pediatric field), so that one can hardly find one's way in this jungle. What is missing is a comprehensive overview of the recent and relevant supportive recommendations and guidelines for everyday clinical practice. There is also a need to change the requirements for SC with regard to new immunological therapy approaches.

In the following we want to provide a clear and recent compilation of the most relevant guidelines and recommendations for SC in childhood leukemia. This overview is intended to enable the clinician to quickly find the right and up-to-date management system for the side-effect at hand. Since new immunological and molecularly targeted therapy methods are on the advance, we not only provide a summary of the current supportive measures for conventional ALL therapies (Table 1), but also an outlook on new therapies already used and their specific side-effects and needs (Table 2). Given the abundance of recommendations and publications on the subject of supportive care in childhood leukemia, we have limited ourselves to the evidence and guidelines that are important and relevant to us.

**Table 1:** Compilation of relevant guidelines and recommendations for supportive measures in conventional ALL therapy.

**Type of supportive care**	**Recommendations**	**Most helpful references for clinical practice**
**Infectious complications during neutropenia**	Fever criteria and vital signs have to be defined and strictly monitored In the case of fever in neutropenia, blood cultures collected from all central venous access devices (possibly also from peripheral vein) Further laboratory tests: peripheral blood cell count (including differential blood count), C-reactive protein, creatinine, transaminases, and venous blood gas analysis; possibly also interleukin 6, procalcitonin First-line antibiotic therapy of fever without a focus as monotherapy or combination therapy possible; preferred first-line betalactam antibiotics are piperacillin-tazobactam (61%), ceftazidime, cefepime and ceftriaxone (but large international and interinstitutional differences) Time to escalation 48 to 72 h, also dependent on individual reassessment Consider local resistance epidemiology	(16) for international differences see references in the text
**Antifungal prophylaxis and treatment**	Prophylaxis: Systemic antifungal prophylaxis recommended for all newly diagnosed and all high-risk ALL patients No routinely used antifungal prophylaxis in low-risk patients Use of mold-active agent No routine use of amphotericin Treatment: Risk-adapted antifungal prophylaxis Antifungal treatment in neutropenic fever: in patients at high-risk for invasive fungal infections start of therapy with liposomal amphotericin B after 72 h of fever; also possible caspofungin, or voriconazole	(16, 17)
**Pneumocystis jirovecii prophylaxis**	First-line: trimethoprim/sulfamethoxazole 2–3 times a week, possibly once a week sufficient in children; duration: whole period at risk Second-line: pentamidine, atovaquone or dapsone	(18)
**Blood product substitution**	All blood products given should be leukocyte-depleted, virus-inactivated or irradiated at 30 Gy. There are no generally applicable guidelines, but a Hb level of <6–8 g/dLcan be used as a benchmark for erythrocyte concentrates (also depending on clinical situation) and platelet levels <10.000/μL can be used as threshold value for platelet transfusions	(19–21)
**Hemostaseologic recommendations**	No evidence-based guidelines in pediatrics Low-molecular-weight heparin is efficient and safe for prophylaxis of thromboembolic complications in children	(22, 23)
**Antiemetic prophylaxis and treatment**	Prophylactic administration of a 5-HT_3_ antagonists (ondansetron, granisetron)	(24)
**Dental care and mucositis**	Dental care: Oral examination before chemotherapy teeth brushing with fluoridated toothpaste flossing and fluoridated rinses chlorhexidine rinses Mucositis: Oral cryotherapy low-level laser recombinant human keratinocyte growth factor-1 sodium bicarbonate and benzydamine rinses	(25–27)
**Acute tumor lysis syndrome**	Prevention: Close patient monitoring for low-risk patients Hydration and allopurinol or rasburicase for intermediate-risk patients Hydration and prophylactic rasburicase in high-risk patients Therapy: Aggressive hydration and diuresis Allopurinol or rasburicase No alkalinization recommended	(28)
**Drug-induced diabetes mellitus** **Hypertriglyceridemia**	Approx. 50% of children with drug-induced hyperglycaemia require insulin treatment (depending on age and weight) Triglycerides >1000 mg/dL: low-fat diet, omega-3 fatty acids and acipimox	(29, 30)
**Osteonecrosis**	Early detection by magnetic resonance imaging beneficial Treatment with bisphosphonates ameliorates pain and mobility No improvement of already destroyed bone structure	(31)

**Table 2:** Compilation of relevant guidelines and recommendations for supportive care in new therapies for childhood ALL.

**Type of supportive care**	**Recommendations**	**References**
**CRS (systemic inflammatory response syndrome)**	Blinatumomab CRS upon starting first infusion; temporarily discontinue treatment or preventive steroid administration CAR T-cell therapy During the first one to two weeks after infusion, toxicity higher; no possibility to reverse or stop the infusion General management of CRS Antipyretics, iv hydration, diagnostics of infection and treatment; oxygen, toxilizumab ± corticosteroids; vasopressors, ICU	(32–35) (Tocilizumab in pediatric ALL)
**Immune effector cell-associated neurotoxicity syndrome (ICAN)**	Close monitoring for cerebral edema for patients with CNS disease or a history of seizures, prophylaxis with levetiracetam is recommended corticosteroids as first-line therapy tocilizumab (2^nd^ line) siltuximab or anakinra (3^rd^ line)	(32, 33)
**Hemophagocytic lymphohistiocytosis after CAR T-cell therapy (car-HLH)**	Corticosteroids as cornerstone treatment cyclosporine or anakinra (2^nd^ line)	(36–38)
**Hypogammaglobulinemia**	Regular monitoring of immunglobulins after immunotherapy is recommended (typically after 1–3 months) IgG replacement iv or sc when IgG <400 mg/dL; level >1,000 mg/d is favorable. Recovery of B-cell function by monitoring peripheral B-cell count and IgG levels. Discontinue replacement therapy when IgG >400 mg/dL is maintained without replacement therapy	(39, 40)

## Supportive care in conventional childhood ALL therapy

### Infectious complications and neutropenia

Chemotherapy-induced neutropenic fever is a common complication in children with ALL. As a consequence, bacterial bloodstream infections constitute the most frequent and life-threatening adverse events (1, 41–43). Factors that are associated with an increased likelihood of infectious complications are chemotherapeutic intensity, neutropenia, comorbidities, and male gender (42, 44). Underweight patients are also reported to have a higher incidence of febrile neutropenia, likely due to their impaired immune response, a consequence of the decreased production of complement factors, cytokins, and immunoglobulins (45–47). In a high percentage of cases of neutropenic fever in ALL it is not possible to determine the focus of the underlying infection by documenting it clinically (e.g., pulmonary infiltration) or microbiologically (positive blood culture) within the first hours or days of fever onset. The standard of care is still the timely start with broad-spectrum antibiotics (42, 48). Although several studies have improved algorithms regarding initiation, modification, and termination of antimicrobial treatment strategies, how to empirically treat the assumed pathogen remains the subject of controversy (43, 49). There are also major international differences in the existing guidelines and recommendations (50–55), starting with the definition of fever and the vital signs to be monitored when a fever occurs in neutropenia to the question of whether to start with a mono- or combination therapy, which antibiotics to choose and how to deal with therapy-resistant fever. Analysis of local resistance patterns and the modification of the antibiotics at the moment of availability of antibiograms is indispensable (42–44, 56). It is recommended that blood culture sampling be obtained from all lumens of a central venous catheter, with the correct volume and proper antiseptic procedure being essential (56, 57). Decision-making regarding the utilization of antibacterial agents should be adjusted to local bacterial resistance patterns and local epidemiology. Frequent clinical examination and precautions against possible fungal superinfections are proposed, while also considering the potentially harmful effects of prolonged antibiotic usage (e.g., Clostridioides difficile infection, invasive fungal disease, drug toxicities, and antibiotic resistance). Granulocyte colony-stimulating factor (G-CSF) may be used in clinically significant neutropenia (58).

### Antifungal prophylaxis and therapy

Children with malignant hematological diseases, especially those with high-risk criteria or myeloablative therapy before stem cell transplantation, have an increased risk of developing an invasive fungal infection (59). Infections with candida and aspergillus are the most common and can significantly increase morbidity and mortality (60). Some publications have summarized guidelines for the systemic prophylaxis of invasive fungal infections in pediatric oncology in the past few years (61, 62), but the most recent work is a practical guide by Lehrnbecher et al. published in 2020 (17). Systemic antifungal prophylaxis is recommended here for all newly diagnosed and all high-risk ALL patients, especially if they are in neutropenia. In the case of low-risk ALL patients, there is no evidence for routine fungal prophylaxis. It is also recommended that a mold-active agent be used (instead of routinely used fluconazole). Routine prophylaxis with amphotericin is not recommended here, but can be useful in some locations (depending on local resistance patterns). It has also been shown that azoles (e.g., fluconazole), in contrast to amphotericin, lead to interactions with chemotherapeutica such as e.g., vincristine (63).

Antifungal treatment in patients at high risk for fungal infections is usually started within 48–72 h of onset of neutropenic fever. Possible substances are liposomal amphotericin B, but also caspofungin or voriconazole (16).

### Pneumocystis prophylaxis

Pneumocystis jirovecii phrophylaxis is one of the topics in supportive care for childhood leukemia, for which there are precisely defined, generally applicable guidelines. These were worked out by the European Conference on Infections in Leukemia (ECIL). The recommendation is to administer trimethoprim/sulfamethoxazole two to three times a week as a first-line medication, over the entire period in which the patient is at risk for developing pneumocystis jirovecii pneumonia (18). In children, a single dose per week may be sufficient (18, 64). Second-line alternatives are pentamidine, atovaquone and dapsone (18, 65).

### Blood transfusion guidelines

Anemia and thrombocytopenia are seen in nearly all children with ALL at some point during their disease course, namely as a result of bone marrow infiltration, chemotherapy, or associated illness. Nutritional deficiencies (e.g., iron, B12, or folate) or blood loss can further contribute to the development of cytopenia (19, 66). The lack of dedicated studies means there are few conclusions, pediatric evidence-based guidelines and criteria for blood product support. As Wong et al. concluded from their survey among pediatric hematology/oncology specialists, transfusion practices vary widely (66). Nonetheless, the following thresholds are most common:

Erythrocyte transfusions are often performed when the hemoglobin concentration is between 6 and 8 g/dL and declining or when the child manifests signs of anemia such as severe fatigue, headache, irritability, or tachycardia. Of note, the patient's individual clinical situation is often more important here than specific transfusion limits (20). Platelet transfusions are indicated for patients with platelet counts <10,000 /μL or for thrombocytopenic patients if there is active bleeding (21, 67, 68). However, it must be mentioned here that some treatment protocols advise platelet transfusions when the platelet count is <30,000/μL during induction therapy. Irradiated/virus-inactivated blood and leuko-depleted products should be used. Patient monitoring is advised, as the likelihood of alloimmunization increases with every additional blood product (15, 19, 66, 69).

### Hemostaseologic recommendations

Thromboembolic complications are well known and frequently observed in childhood ALL (70) and can lead to acute events with increased morbidity and mortality as well as to long-term sequelae. However, there is no consensus on the safety and effectiveness of thromboembolic prophylaxis in children to date and the most varied approaches from different centers were observed (71). So far, many studies have dealt with this topic, with the result that only low molecular weight heparin can effectively and safely reduce thromboembolic complications in pediatric oncology (especially in ALL) (22, 72). A significant statement on thrombosis prophylaxis using other therapies such as antithrombin replacement or vitamin K antagonists has not yet been made (22). There is a need for large, prospective, randomized studies that compare various prophylactic measures. A randomized controlled study is currently running on the subject of thromboprophylaxis in childhood ALL with low molecular weight heparin (73). A prospective randomized study by Greiner et al. recommends thromboprophylaxis with enoxaparin for children and adolescents with ALL during induction therapy (23). Another randomized controlled study explores the effectiveness of apixaban compared to standard of care (no anticoagulation) for prevention of venous thromboembolic events in childhood ALL (74).

On the other hand, recently published guidelines from the American Society of Hematology provide clear recommendations for managing thrombotic complications in adult cancer patients (75).

### Nausea and vomiting

Our knowledge of the perfect remedy for nausea and vomiting is still incomplete. It is probably also due to the fact that these symptoms can be very different in individual cases and therefore a patient-specific approach to therapy is certainly sensible. An algorithm for assessing the emetogenicity of chemotherapy and possible therapy was developed by Dupuis et al. (76). In general, the use of selective 5-HT_3_ antagonists (granisetron, ondansetron) is recommended for the prophylaxis and therapy of chemotherapy-induced vomiting. This therapy is highly effective in most patients with ALL. If the effect is not sufficient, a combination therapy with corticosteroids or cannabinoids is possible [reviewed in Phillips et al. (24)]. Aprepitant in combination with standard antiemetics is another possibility for treating therapy-resistant nausea in pediatric oncology and is well tolerated (77).

### Dental care and mucositis

Oral health care is a very important topic in childhood malignancies (25). An oral examination is recommended for every child in need of cytostatic therapy for malignant disease. Collaboration with pediatric dentists is desirable to best complete dental treatment before start of immunosuppressive therapy. During cancer therapy it is particularly important to pay attention to caries prevention by brushing the teeth and tongue twice a day with a soft-bristled nylon toothbrush and fluoridated toothpaste. Dental floss is also recommended. Fluoridated mouth rinses and chlorhexidine for plaque-induced gingivitis or periodontal disease should be prescribed (26, 78).

If mucositis occurs as a side-effect of cytostatic therapy, it can be treated in various ways (depending on the severity): oral cryotherapy, laser therapy (low-level), recombinant human keratinocyte growth factor-1, sodium bicarbonate and benzydamine rinses are evidence-based measures for the management of oral mucositis (27). In addition, systemic analgesic medication is often required.

### Tumor lysis syndrome

The tumor lysis syndrome is the most common emergency situation that occurs in children with ALL, especially at the beginning of cytostatic therapy (79–81). Hyperkalemia, hyperuricemia, hyperphosphatemia, and hypocalcemia can occur either spontaneously as a result of the leukemia itself or as a result of the therapeutic effects. If left untreated, these metabolic and electrolyte imbalances lead relatively quickly to seizures, cardiac arrhythmias, kidney failure, multiple-organ failure, and death. For this reason, general guidelines for the therapy and prophylaxis of tumor lysis syndrome were drawn up a decade ago and are still valid today (28, 80): for prevention of tumor lysis syndrome it is recommended that patients be hydrated and receive allopurinol (intermediate-risk) or rasburicase (high-risk patients). In low-risk patients close monitoring may be sufficient. Therapy of a manifest tumor lysis syndrome requires aggressive hydration for diuresis induction and treatment with allopurinol or rasburicase (alkalinization not recommended).

### Hypertension

Hypertension in pediatric ALL patients during treatment is a well-known side-effect, but data on the incidence and prevalence are limited. As the prevalence of hypertension in the healthy pediatric population is approximately 3.5% (82), children and adolescents with ALL develop hypertension in 12–45% during induction therapy (83–87). Nevertheless, there are no uniform guidelines for the treatment of hypertension during treatment for childhood ALL. A helpful review on this topic was written by Murphy et al. (88), in which an algorithm for the diagnosis and therapy of hypertension in pediatric ALL is proposed.

### Neurotoxicity

Neurotoxicity during conventional childhood ALL therapy can affect both the central and the peripheral nervous system [reviewed in Schmiegelow et al. (89)]. It shows a wide spectrum of manifestations and can be caused by the use of methotrexate, intrathecal chemotherapy and also by corticosteroids and occurs in about 10–15% of patients with childhood ALL (90–96). Seizures can occur in up to 10% of ALL patients during intensive chemotherapy (94). They are either an isolated symptom or are secondary to infections, electrolyte imbalances or metabolic disturbances. They can also occur as an accompanying symptom of cerebral toxicity, for example in the context of cerebral hemorrhage, thrombosis, methotrexate-associated toxicity or posterior reversible encephalopathy syndrome (PRES). Some patients require prolonged anticonvulsive therapy, with female gender being a risk factor (97). Methotrexate-associated toxicity seems to be promoted by the presence of a vitamin B12 deficiency (98) and depends on the therapeutic regimen, dose and co-administration of other substances (90). Additionally, small case studies have shown that dextromethorphan or aminophyllines may be helpful in methotrexate-induced toxicity (99, 100).

Some patients develop psychoses undergoing therapy with corticosteroids during ALL treatment (92). Sleep medication, tranquilizers or anti-psychotic medication may then be required (101).

Peripheral neuropathy (motoric or sensory) may occur in pediatric ALL patients during treatment with vincristine, mostly reversible (102, 103). Interaction with certain medications, such as azoles, can increase this neurotoxicity (104). Strategies to manage peripheral neuropathy are still not proven and fail to relieve symptoms (105).

### Metabolic alterations (diabetes mellitus, hypertriglyceridemia)

The frequent use of glucocorticoids requires monitoring of several side-effects in supportive care. The most common complication is drug-induced diabetes mellitus (DIDM), with a wide reported prevalence of between 9.7 and 69% in pediatric ALL patients (29). Despite the complications of DIDM during ALL therapy, which include diabetic ketoacidosis, a higher risk for infections, and a higher incidence of febrile neutropenia, the literature on its management is sparse, and there is a lack of standard guidelines or recommendations for the treatment of impaired glucose tolerance (29, 106, 107).

Another very common metabolic phenomenon is the occurrence of asparaginase-induced hyperlipidemias, especially hypertriglyceridemias, during the induction and reinduction phases of ALL therapy (30). Although these hypertriglyceridemias usually have few side-effects and are transient, they must be noticed and observed and, in rare cases, treated.

### Osteonecrosis

Osteonecrosis is a common complication of ALL therapy, especially in adolescents, and often significantly affects quality of life of the affected patients. Early detection in asymptomatic patients using magnetic resonance imaging to examine bone vascularization and structure is beneficial (108, 109). The management of osteonecrosis in childhood ALL is risk-stratified and ranges from observation and adaptation of therapy with cytostatics and corticosteroids to pharmaceutical and surgical approaches (110). It has been shown that therapy with bisphosphonates (combined with dietary calcium, vitamin D and physical therapy) was able to reduce pain and thus increase mobility, but not stop the destruction of joints (31, 111). In addition, new conservative therapeutic methods such as hyperbaric oxygen therapy have been used further to ameliorate the outcome (108).

### Cardiotoxicity

It is evident that cancer treatment, particularly therapeutic regimens that include cumulative doses of anthracyclines, can have a cardiotoxic profile. The risk for cardiovascular problems varies greatly depending on the type and dose of anthracycline. However, anthracycline-induced heart failure may take years or even decades to manifest, although signs of cardiac dysfunction can be observed before symptoms appear. Already early after chemotherapy diastolic dysfunction and regional systolic impairment may appear. Therefore, cardiovascular monitoring, prevention, and early detection of cardiac dysfunction are of the utmost importance (112).

### Pain management

Adequate pain management in children with ALL is an indispensable supportive care tool. In these patients, pain often occurs in connection with osteonecrosis, peripheral neuropathy caused by vincristine or mucosal or gastrointestinal side-effects (e.g., mucositis, enteritis, obstipation) and must be administered in the correct dose for the respective indication, regardless of the substances' possible off-label use in children (113). Going into detail about pain medication in pediatric oncology would exceed the scope of this review. Nevertheless, it has to be mentioned here as an important supportive topic.

## Supportive care in selected new therapies for childhood ALL

In the following compilation of supportive measures for new therapies for childhood ALL, we focus on selected therapies, i.e., the tyrosine kinase inhibitor (TKI) imatinib and the immunological therapeutics blinatumomab and CAR T-cells:

### Resistance and intolerance to TKIs

The TKI imatinib is generally well tolerated, and the risk for severe adverse effects is low. Intolerance is mainly due to the off-target activity of TKIs, as they act in cancerous and non-cancerous cells. Adverse effects most commonly include mild to moderate edema, nausea and vomiting, diarrhea, muscle cramps, and cutaneous reactions. Hepatic transaminase level elevations and myelosuppression occur less frequently and resolve with interruption of therapy (114, 115). In the field of pediatrics, the most important potential side-effects include perturbations in bone metabolism. There are single case reports in prepubertal children that have demonstrated massive growth retardation. Evidence from adult studies suggests that imatinib causes hypocalcemia and hypophosphatemia. Despite the efforts to study the effects of TKIs in pediatric patients, the etiology and cause of growth retardation remain obscure (116). However, as general recommendations are missing and as long-term treatment with imatinib is associated with low bone-mineral density, regular monitoring and assessment of adequate vitamin D, calcium, and phosphate intake seem crucial. However, it must be mentioned here that patients with Ph-positive ALL are usually not affected by growth retardation because they, unlike e.g., patients with chronic myelogenous leukemia, only take TKIs for a limited time.

### Cytokine release syndrome

CRS, a systemic inflammatory response syndrome, is probably the most threatening side-effect of both blinatumomab and CAR T-cell therapies. Though frequently reported, its pathophysiology is still poorly understood. CRS is mainly ascribed to a supraphysiologic release of a broad spectrum of pro-inflammatory cytokines, such as tumor necrosis factor alpha, (TNF-α), interleukin-2 (IL-2), and interferon-gamma (IFN-y), that can activate the monocyte/macrophage system, consequently triggering the production of a wide array of other pro-inflammatory proteins and result in increased C-reactive protein (CRP) levels and sometimes hyperferritinemia. In response to T-cell activation, symptoms may vary from myalgia, low-grade fever, and headache, hours to several days after the infusion, to high fevers, sinus tachycardia, hypotension, or hypoxia. Generalized endothelial activation and capillary leak syndrome may further result in pulmonary edema, respiratory failure, cardiogenic shock, or even multiorgan failure. Additionally, activation of the complement system may lead to a procoagulant state and macrophage activation syndrome (13, 32, 33, 117, 118).

Although both CAR T-cells and blinatumomab evoke similar effects, there are major differences in clinical presentation, grading, and management of CRS. Blinatumomab therapy may trigger CRS upon starting the first infusion, dose escalation, or after restarting the infusion. Given the short half-life of blinatumomab, CRS progression might be suppressed by temporarily discontinuing the treatment, or preventive steroid administration. Conversely, CAR T-cell therapy-related CRS usually occurs during the first 1 to 2 weeks after infusion and the degree of toxicity is reportedly higher. Once administered, the CAR T-cells are engineered to persist, resulting in no possibility to reverse or stop the infusion (32, 119).

In order to mitigate possible life-threatening sequelae, early diagnosis and prompt management of CRS seem crucial. Mahadeo et al. published pediatric-specific guidelines (34) for CRS grading. In terms of predictive laboratory markers, baseline thrombocytopenia, and baseline elevated markers of endothelial activation, such as angiopoietin-2 (ANG2) and von Willebrand factor (vWF), have been associated with the development of severe CRS (32, 119).

In the case of prolonged CRS or conventional therapies failure (e.g., IV fluids, vasopressors), IL-6-directed therapy or corticosteroids are recommended to terminate the CRS cascade. Corticosteroids have established efficacy in treating CRS; nonetheless, their use should be carefully considered because of the risk of suppressing CAR T-cell expansion. The administration of tocilizumab has in many cases resulted in the rapid resolution of hemodynamic instability and attenuation of symptoms within 4 hours. This monoclonal anti-interleukein-6 receptor antibody was approved for the treatment of children and should be administered in moderate to severe CRS cases (35). However, many cases of severe CRS may require additional immunosuppression with corticosteroids (13, 32, 33, 119).

### Immune effector cell-associated neurotoxicity syndrome

Neurologic sequelae of CAR T-cells and blinatumomab are commonly referred to as immune effector cell-associated neurotoxicity (ICAN), and include clinical symptoms of toxic encephalopathy with delirium, seizures, or cerebral edema. Initial manifestations in children can be subtle, notably inattention, headache, dizziness, irritability, or tremor. Cases of impaired speech or writing and asterixis are also reported (13, 32, 36, 117). Despite the overlapping time of onset with CRS, ICAN can proceed biphasically. The exact pathogenesis is still poorly understood; however, evidence implies that severe neurotoxicity occurs almost exclusively in patients who have developed CRS before. Presumably, endothelial activation due to the supraphysiologic cytokine release results in increased permeability of the blood-brain barrier to neurotoxic cytokines (12, 120, 121).

Similar to the management of CRS, the anti-IL-6 therapy is also recommended for neurologic sequelae, with evidence showing better outcomes in patients concurrently exhibiting symptoms of ICAN and CRS. However, tocilizumab proved to have limited efficacy in resolving neurologic toxicity, possibly because CAR T-cells and inflammatory cytokines are known for better penetration of the blood-brain barrier than is tocilizumab. For patients with CNS disease or a history of seizures, anti-seizure prophylaxis with levetiracetam is recommended. Patients should be closely monitored for signs and symptoms of cerebral edema and, as ICAN is also reported to occur as a late complication, given appropriate education before being discharged from the hospital (12, 32, 33, 120, 121).

### Hemophagocytic lymphohistiocytosis after CAR T-cell therapy (car-HLH)

Evidence suggests that human monocytes are the main source of IL-1 and IL-6 in CRS. Thus, severe CRS shares clinical features with macrophage activation syndrome, including fever, hyperferritinemia, and multiple-organ dysfunction. Cases of fulminant HLH as a variant of conventional CRS, characterized by severe immune activation, lymphohistiocytic tissue infiltration, and immune-mediated multiorgan failure, have also been reported (36–38). As symptoms can manifest similarly to severe sepsis or CRS, diagnosis of HLH requires experienced clinical judgment. Effective treatment of HLH includes aggressive immunosuppression, with corticosteroids remaining the cornerstone treatment. However, over half of patients may be steroid-resistant. Thus, the addition of cyclosporine (CSA) or anakinra, an IL-1 antagonist, may be required.

### Hypogammaglobulinemia

As the potent ability of anti-CD19 CAR T-cells and blinatumomab to target malignant CD19-expressing B-cells also results in the destruction of normal B-cells, most patients develop hypogammaglobulinemia following immunotherapy (122). This results in decreased production of antibodies and exposes the patients to an increased risk for potentially life-threatening infections. It has been demonstrated that CAR T-cells can persist for years in patients and long-term B-cell aplasia can occur even after complete remission (123, 124). Hence, regular monitoring and substitution of immunoglobulins is therefore recommended after immunoglobulins have dropped to a level below 400 mg/dL (39). Patients can be transitioned from intravenous to subcutaneous immunoglobulin replacement to maintain an IgG level >1,000 mg/dL (40). Noteably, differences in hypogammaglobulinemia were found in children as compared to adults (125).

### Infectious complications combined with new therapies

This topic is very extensive and would exceed the scope of this review. It should only be mentioned here that infectious complications that were caused by previous conventional therapy regimes are expanded to include the spectrum of side-effects of the new therapies. In addition to cytotoxic treatment, new immunological therapies expose patients to an additional risk of serious infections by triggering CRS (often indistinguishable from sepsis) or hypogammaglobulinemia. Therapies with corticosteroids or toxilizumab additionally increase the potential for developing serious infectious side-effects (126).

### Long-term toxicities after new therapies

In the case of therapy for malignancy in childhood or adolescence, it is always important to pay attention to the long-term effects of the used substances and to monitor them precisely as they may affect the patient for the rest of their life. New immunological therapies against ALL can not only have acute side-effects (e.g., CRS, ICAN, HLH), but also trigger long-term effects in a child's growing organism. There are not only effects on the immune system (e.g., hypogammaglobulinemia), but also long-term toxicities affecting other organ systems. Cardiac, pulmonary, ophthalmological and renal toxicities are already known and described (127). An increased risk of secondary malignancies has not yet been observed after CAR T-cell therapy, but the use of lenti- and retroviral vectors for their production could be viewed as critical (128).

## General important supportive domains in the field of ALL

An equally important area of supportive care comprises the management of psychological issues. Childhood cancer patients and their parents will experience significant psychological distress throughout the course of the disease, indicating that psychosocial support for both the children and their families is crucial in mitigating symptoms of distress. There is broad agreement that patient-reported outcome measurements can be beneficial for early recognition of clinical and psychological effects that the disease and its treatments have on patients' and their families' lives. We recently demonstrated that daily symptom monitoring in children with cancer affords the opportunity to identify symptoms early and initiate appropriate clinical reactions (129, 130).

Nutritional status is another outcome measurement factor influencing treatment tolerance and overall quality of life. Chemotherapy-induced nausea/vomiting, anemia, and fatigue can aggravate poor nutrition, consequently worsening fatigue (46, 131). Furthermore, undernutrition, overweight, and obesity alter drug pharmacokinetics, resulting in increased toxicity and vulnerability to overdose. For instance, high-dose MTX has delayed clearance in underweight patients, causing acute kidney injury or hepatotoxicity (132, 133). Nutritional strategies should be considered and integrated as fundamentals of pediatric oncology, to adequately provide macronutrients, beneficial components like omega-3-polyunsaturated fatty acids, or micronutrients such as vitamin D (134).

## Conclusion

In response to a need for a summary of the flood of guidelines and recommendations on the subject of supportive care in childhood leukemia, we have tried to give a concise and recent overview of the existing clinically relevant literature. The supportive measures will be gradually expanded to include measures that are unavoidable in combination with new molecular and immunological therapies for ALL.

## Data availability statement

The original contributions presented in the study are included in the article/supplementary material, further inquiries can be directed to the corresponding author/s.

## Author contributions

Literature search, data collection, and data interpretation were performed by AP, CS, RC, and MM. The first draft of the manuscript was written by AP and CS. All authors contributed to the conception and design of this review, commented on previous versions of the manuscript, and read and approved the final manuscript.

## Conflict of interest

The authors declare that the research was conducted in the absence of any commercial or financial relationships that could be construed as a potential conflict of interest.

## Publisher's note

All claims expressed in this article are solely those of the authors and do not necessarily represent those of their affiliated organizations, or those of the publisher, the editors and the reviewers. Any product that may be evaluated in this article, or claim that may be made by its manufacturer, is not guaranteed or endorsed by the publisher.
